# Avian influenza virus cross‐infections as test case for pandemic preparedness: From epidemiological hazard models to sequence‐based early viral warning systems

**DOI:** 10.1111/1751-7915.14389

**Published:** 2024-01-16

**Authors:** Harald Brüssow

**Affiliations:** ^1^ Division of Animal and Human Health Engineering, Department of Biosystems KU Leuven Leuven Belgium

## Abstract

Pandemic preparedness starts with an early warning system of viruses with a pandemic potential. Based on information collected in a multitude of surveys, hazard models were developed identifying influenza viruses presenting a pandemic threat. Scores are attributed for 10 viral traits by expert panels which identified avian influenza viruses (AIV) belonging to subtypes H7N9 and H5N1 as representing the greatest pandemic risk. In 2013, more than 100 human cases infected with AIV H7N9 were observed in China. Case fatality rate (CFR) was high (27%), but the human‐to‐human transmission rate was low and by serological evidence H7N9 did not spread widely. Nevertheless, until 2019 more than 1500 H7N9 patients were identified characterized by a high CFR of 39%. Serology demonstrated that mild infections with H7N9 were widespread. In 2003, more than 400 people experienced AIV H7N7 cross‐infection causing mainly conjunctivitis during a large poultry epidemic in The Netherlands. Between 1996 and 2019, a total of 881 human infections with avian H5N1 viruses were documented showing a CFR of 52%. Outbreaks were centred on South East Asia and showed close associations with epizootics in poultry. Mutations predisposing to human cross‐infections were identified in the haemagglutinin (HA) and the RNA polymerase subunit PB2 of human H7N9 isolates. Human H5N1 isolates showed mutations in the receptor binding domain of HA and transmission in mammals could be obtained by as few as four additional aa changes introduced experimentally. Researchers have defined viral point mutations in HA, PB2 and the nucleoprotein NP that allowed AIV to cross the species barrier to mammals with respect to receptor recognition, RNA replication and escape from innate immunity respectively. Based on this insight a sequence‐based early warning system for AIV preadapted to human transmission could be envisioned. Mink farms and live poultry markets are prime targets for such sequencing efforts.

## PANDEMIC PREPAREDNESS

The importance of pandemic preparedness or the lack of it was critically underlined during the recent COVID‐19 pandemic. Pandemic preparedness is a complex task and requires the collaboration of many scientific disciplines and the coordination of different public health measures under governmental guidance. In a first approximation, pandemic preparedness can be differentiated into two major groups of tasks. The first group of activities encompasses the monitoring of a prevailing infection situation and strives to develop tools for an early viral warning system assessing the zoonosis potential of animal viruses to cause a pandemic. A virus‐centred approach is justified by the observation that all pandemics of the last century were cross‐species infections with animal viruses. However, many microbes can represent pandemic threats and soon antibiotic‐resistant bacterial pathogens might represent a major challenge for medical practice. The second group of activities in pandemic preparedness concerns the coordination of efficient control measures once a novel infection is spreading and taking epidemic dimensions. The current contribution will deal with the first aspect namely the establishment of an early viral warning system for avian influenza virus (AIV) cross‐infections into the human population. In the first part, standard hazard models will be presented and the epidemiology of AIV cross‐infections in humans will be reviewed. In the second part, it is asked whether a sequence‐based risk assessment system for AIV genomes should supplement these efforts.

## HAZARD MODELS

The US Centers for Disease Control and Prevention (CDC) and the World Health Organization (WHO) have developed an influenza risk assessment tool (IRAT) (Burke & Trock, 2018) and tools for influenza pandemic risk assessment (TIPRA) respectively. These early warning tools are based on similar principles and represent a complex integration of information contributed by experts from a large number of different scientific disciplines. The WHO document subdivides the early warning tool into three subtasks. The first requires the identification of novel human infections by clinicians and epidemiologists, the assessment of the disease severity and the evaluation of the population immunity level towards the new virus which will determine the likelihood of the viral spread and the degree of its clinical impact on infected subjects. This activity has to be complemented by the work of scientists involved in a second subtask, that of animal health. Veterinarians, epidemiologists and ecologists should determine the spatial and temporal spread of viral infections in animals and the specific ecological conditions favouring the exposure of humans to these animal viruses. The third subtask involves the work of virologists who study the receptor binding properties of these viruses, their host range and transmission in relevant mammalian models, and the identification of viral genome characteristics associated with these traits. More applied virologists will then investigate the susceptibility of these viruses to antiviral treatment options and the development of fitting vaccines. This latter task is an important public health measure to contain a beginning epidemic, once transmission of a novel virus has occurred in a larger part of the human population.

IRAT and TIPRA are hazard models evaluating the risk of sustained human‐to‐human transmission of a given influenza virus after analysing 10 risk elements. They include properties of the virus, antiviral immunity and resistance attributes in the human population, and elements of animal virus ecology. Scores ranging from 1 to 10 representing increasing zoonosis/pandemic risk are attributed by an expert panel based on available, sometimes however incomplete data sets, for each of the 10 risk criteria. A summary score for each investigated influenza virus is consolidated in an expert meeting. IRAT from CDC differentiates the scores along two axes namely according to emergence (species jump) and to impact (disease severity and epidemic spread) of candidate animal viruses. When IRAT was run in 2018 for 14 animal influenza viruses, highest scores of about 7 for both emergence and impact were attributed to H7N9 followed by H5N1 and H5N6 avian influenza A virus strains for impact. The high scores for these two types of influenza A viruses were based on a multitude of observations which will here be summarized, starting with data on H7N9 influenza A viruses.

## H7N9 INFECTIONS

### The 2013 outbreak in China

Chinese virologists reported in 2013 three cases of fatal lung infections with AIV H7N9 in unlinked patients from Shanghai and Anhui. Two had exposure to birds by working at or visiting live bird markets before falling ill. All three patients were infected with closely related H7N9 virus strains sharing greater than 97.7% sequence identity over the eight genes composing the segmented viral genome of influenza viruses. All genes were of avian influenza virus origin. The isolates represented a rearrangement among three avian viruses. The haemagglutinin (HA) gene H7 was derived from a duck virus, the neuraminidase (NA) gene N9 was derived from viruses widely circulating in wild birds, while the remainder of the genes were derived from a H9N2 virus circulating in bramblings, a passerine bird from the finch family (Gao, Cao, et al., 2013). The genomes of the three viruses were sequenced to identify potential markers for an avian‐human cross‐species infection and for virulence attributes explaining the deadly clinical outcome in the three patients. The researchers identified a Q226L (glutamine in one letter code at amino acid (aa) position 226 replaced by leucine) substitution in the HA protein which was associated with binding to α‐2,6 linked sialyl glycans which are found on human cells and are used as viral receptor. This mutation might also increase the ability of the virus to be transmitted by air. In addition, a deletion of five aa in the viral NA stalk was observed that was previously described to change viral tropism to the respiratory tract and to increase its transmission in domestic poultry. Finally, the E627K substitution in the viral RNA polymerase subunit PB2 might have increased its replication in mammalian cells (Gao, Cao, et al., 2013). The clinical observations in subsequent 111 Chinese patients hospitalized with a laboratory‐confirmed H7N9 diagnosis were summarized (Gao, Lu, et al., 2013). Overall, 77% were admitted to an intensive care unit (ICU) and 27% died; 30 surviving patients remained hospitalized mostly in ICU and only 49 patients were discharged after full recovery. The patients were predominantly older than 60 years and male. Multivariate analysis identified an underlying disease as risk factor for death. Presenting symptoms were fever and cough, sometimes combined with diarrhoea. Pneumonia developed in all of them. Complications were acute respiratory stress syndrome (71%) and shock (26%). Antiviral treatment with oseltamivir was initiated in all patients and—when successful—time to viral clearance was 6 days (Gao, Lu, et al., 2013). The Chinese researchers conducted an epidemiological study to characterize the 2013 H7N9 epidemic in China. Overall, 1372 patients were hospitalized in 2013 with pneumonia of unexplained origin; 7.6% were confirmed to be H7N9 infections. Cases occurred in 10 provinces but most cases occurred in the geographically adjacent Zhejiang, Shanghai and Jiangsu provinces. The researchers distinguished clear phases: sporadic cases until March, an epidemic peak in April, a declining phase in May 2013 followed by a sharp reduction in cases after the closure of live poultry markets. Only 6% of cases were poultry workers, but 82% of the patients had exposure to chickens on live animal markets. Four clusters of cases were observed consisting each of two family members where one member without known animal contact took care of the diseased family member, suggesting person‐to‐person infection transmission upon close, unprotected contact. An additional 2675 further close contacts were evaluated; 1% developed respiratory symptoms 1 week after contact, but none had a H7N9 infection. Of about 2 million respiratory specimens from outpatients with an influenza‐like illness obtained during a surveillance program in 2013 from China, only eight tested positive for the H7N9 virus indicating that the avian virus did not widely spread through the population (Li et al., 2014).

### Subsequent development in China

Between 2013 and 2019, a total of 1568 cases of human infections associated with H7N9 were documented. The case fatality rate was 39%. All cases were from China and all cases reported exposure to poultry. H7N9 case patients were old (40% were older than 60 years) and mostly male (70%). Since 2014, a seasonal pattern with winter outbreaks of H7N9 virus was observed every year (Philippon et al., 2020). Between Spring 2013 and Spring 2015, Chinese epidemiologists identified 22 clusters of human infections with H7N9 virus. Clusters could result from person‐to‐person transmission or exposure to a shared infectious source. To differentiate between these two possibilities, the researchers computed an incubation period‐weighted score for exposure to the index case patient based on known incubation times. Based on this criterion, 12 contacts of case patients were rated as likely human‐to‐human infection transmissions while six contacts likely reflected infection from a shared source. One case of nosocomial infection was identified in a patient sharing a hospital ward with a case patient (Liu et al., 2017). These data suggest limited transmission of H7N9 infections between humans. Another Chinese study investigated specific serum antibodies to avian influenza viruses H7N9 and H5N1 in poultry market workers from China at two time points in 2013 and compared the data to the seroprevalence of these antibodies in the general population. High antibody titres were found against H7N9 in 7% of the market workers in May 2013 and in 15% in December 2013, half of the workers studied at both time points showed a seroconversion to H7N9 virus. Nasal swabs from poultry market workers were negative for H7N9 by PCR and no worker recalled an influenza‐like disease suggesting asymptomatic infections. Workers with a previous seasonal influenza vaccination showed a significantly lower probability of having elevated serum antibody titres to H7N9 suggesting a partial protection by the vaccine extending to the H7N9 virus, which was not included in the vaccine cocktail. Seroprevalence against H5N1 virus was low (0.8%) in poultry workers. None of the 800 serum samples from the general population showed antibodies against H7N9 or H5N1 (Wang et al., 2014). The data indicate that clinically mild cross‐species infection with H7N9, but not with H5N1 occurred in a number of people exposed to poultry. When using a larger number of sera, that is, 5000 sera drawn in 2014 in Guangzhou/China, two sera tested positive for H7N9‐specific antibodies by neutralization assays. From this prevalence, the researchers estimated that 60,000 infections with H7N9 occurred in Guangzhou in 2014 (Lin et al., 2016). Since in this time period, 16 severe infections and 11 deaths were reported for H7N9 infections in Guangzhou (Chen et al., 2013; Chen, Liu, et al., 2014), the vast majority of H7N9 infections in the general population must have been mild or asymptomatic.

### Events in poultry

That live poultry markets and thus animal‐to‐human cross‐infections are the drivers of the H7N9 outbreak in China was demonstrated by the effect of the closure of these markets. There were 85 confirmed influenza A(H7N9) cases in Shanghai, Hangzhou, Huzhou and Nanjing. Case numbers fell rapidly after the closure and disinfection of the live poultry markets in these cities. The researchers calculated a 97% reduced risk of human infections with H7N9 through live poultry market closure (Yu et al., 2014). In 2017, a bivalent inactivated veterinary vaccine against H7N9 and H5N1 was implemented in China which reduced the seropositivity rate in poultry to these two viruses by more than 90%. Notably, only three human H7N9 cases were reported since the introduction of this veterinary vaccine (Zeng et al., 2018).

The HA protein from H7N9 viruses circulating between 2013 and 2016 did not contain a poly‐basic cleavage site, the defining trait differentiating low (LPAI) from highly pathogenic avian influenza (HPAI) viruses. This concurs with the lack of severe disease in H7N9‐infected chickens and ducks in China during this period but contrasts with the high case fatality observed in human cases during this time period. In 2017, HPAI H7N9 strains emerged in poultry from China (Neumann et al., 2021).

### Molecular analysis

Japanese researchers characterized the human H7N9 isolates from Anhui and Shanghai of the 2013 outbreak. The human isolates replicated efficiently in cultured mammalian cells. In mice, they were substantially more pathogenic than avian H7N9 isolates or human seasonal H1N1 isolates causing severe lung infections. Infected ferrets lost appetite and showed higher nasal than lung viral titres. Only a third of ferrets exposed to airborne droplets from infected ferrets experienced an infection. In macaques, the human H7N9 isolates induced fever and replicated efficiently in the upper and lower respiratory tract and caused a strong inflammatory response. Signs of disease were not observed in infected pigs, chickens and quails. In vitro binding experiments showed a binding of the human H7N9 isolates to α‐2,6‐linked sialyl glycans. The isolates were susceptible to neuraminidase inhibitors such as oseltamivir (Watanabe et al., 2013). Dutch researchers reported that the human H7N9 isolates are transmitted to 75% of contact ferrets by the respiratory droplet route, in contrast to other avian influenza viruses which are not airborne transmitted in ferrets. However, the transmission was less robust with fewer animals becoming infected, and with less and delayed virus shedding compared to seasonal H1N1 virus transmission. Serial airborne transmission of H7N9 occurred in only a quarter of ferret pairs (Richard et al., 2013).

## H7N7 INFECTIONS

Until 1997, only four cases of direct avian‐to‐human transmission of influenza viruses had been described and all involved H7N7 infections (Neumann et al., 2021). In March 2003, a HPAI H7N7 virus spread to 255 poultry farms in The Netherlands leading to the culling of 30 million chickens. By June 2003, a total of 453 people exposed to the H7 avian virus reported symptoms, mostly conjunctivitis (77%) followed by influenza‐like illness (20%). An exposed veterinarian developed pneumonia, died and showed an influenza A/H7 virus in autopsy material. Overall, 8% of exposed people on the farms reported conjunctivitis. The epidemic of H7‐associated human cases showed two temporal peaks going in parallel with the spread of the poultry epizootic (animal epidemic) to other geographical regions in The Netherlands. Three household contacts developed a H7‐associated conjunctivitis indicating a limited human‐to‐human transmission of this AIV. Virus detection was mostly positive from eye swabs during the first 3 days of symptomatic disease. The virus showed a fourfold reduced sensitivity to oseltamivir antiviral. Public health measures were imposed 1 week after the first human infection had been confirmed. However, at that moment, the infection had already spread to about 1000 persons over the country and even abroad. These data demonstrate the importance of movement restrictions for early infection control but at the same time the difficulty to restrict an epidemic to its geographical origin (Koopmans et al., 2004). Notably, the H7N7 genome sequences from the human conjunctivitis cases did not differ significantly from the viruses isolated from chicken on the farms while the H7N7 isolate from the single fatal human infection differed by 14 amino acids substitutions distributed over the PB2, HA and NA genes from the avian outbreak virus (Fouchier et al., 2004).

## OTHER HUMAN INFECTIONS WITH AIV

Human infections with other avian H7 influenza viruses were also reported. An outbreak with a high‐pathogenicity avian influenza (HPAI) virus H7N3 in poultry associated with a 25% death rate in younger poultry flocks led to the culling of 19 million animals in British Columbia/Canada. Two workers involved in the culling developed conjunctivitis and coryza after direct eye exposure (Hirst et al., 2004). Likewise, a small outbreak with a low‐pathogenicity avian influenza (LPAI) virus H7N3 AIV in the UK in 2006 led to a cross‐infection in a poultry farm worker who experienced conjunctivitis (Nguyen‐Van‐Tam et al., 2006). Serological surveys were conducted in Northern Italy among 800 poultry farm workers during avian epizootics with HPAI virus H7N1 between 1999 and 2002 and among 185 poultry workers during an outbreak with LPAI H7N3 in 2003. No worker tested positive during the HPAI outbreaks, while 3.8% were seropositive against H7N3 following the LPAI outbreak in 2003. Conjunctivitis was the main symptom reported with LPAI infection (Puzelli et al., 2005). Other subtypes of AIV were detected in a handful of H9N2 infections in China; all patients recovered uneventfully. Two cases with H10N7 infections were seen in Egypt and Australia, both showed only mild symptoms (Neumann et al., 2021). Single cases of infection with H10 avian influenza viruses were also reported from China. A 73‐year‐old women died from H10N8 infection after visiting a live poultry market. Close contacts were not infected. The isolate was a reassortant AIV with a HA gene from a Eurasian and a NA gene from a North American avian virus lineage. All internal genes were derived from the avian influenza A virus H9N2 that circulates in poultry from China. The HA showed an avian‐like receptor binding preference while the internal PB2 protein had an aa substitution associated with mammalian adaptation (Chen, Liu, et al., 2014; Chen, Yuan, et al., 2014).

## H5N1 INFECTIONS

### Hong Kong

Between 1996 and 2019, a total of 881 human infections with H5N1 AIVs were documented showing a case fatality rate (CFR) of 52% (Philippon et al., 2020). The first outbreak of H5N1 virus in humans occurred in Hong Kong in 1997. The case of a 3‐year‐old boy was the first transmission of a wholly avian influenza virus to a human with a fatal outcome. Infections were detected in 17 further individuals, and in five, the infection was fatal. Transmission was directly from chickens and the outbreak stopped after culling more than 1.5 million chickens (Chan, 2002). Clusters of infection with AIV A/H5N1 viruses were subsequently reported from Vietnam, Thailand, Indonesia, China, Azerbaijan and Turkey.

### South East Asia

Outbreaks were focused on South East Asia with extensions to Turkey and Egypt, all countries with high domesticated poultry populations (Hill et al., 2015). Human cases followed mostly epizootics with H5N1 AIV in poultry. Since 2003, H5N1 outbreaks in poultry have occurred throughout Indonesia. In 2005, three clusters of H5N1 infections were also observed in humans. One consisted of a father and his two young girls in a suburb of Jakarta who all died. No contact with poultry was noted. Another cluster comprised an aunt and her nephew who lived temporarily together, the aunt died from pneumonia. A chicken died in the neighbourhood and the aunt used H5N1‐positive chicken faeces as fertilizer in her garden. A third cluster consisted of a young adult and two children relatives living in rural Sumatra where backyard chickens started to die in the village. All three patients were hospitalized and survived. Contact tracing from all three clusters showed no onward transmission of the H5N1 virus. Overall mortality was 50%. Subsequently, a large outbreak of H5N1 infections occurred in Sumatra in 2006. Sequencing of the isolated viruses showed that all genes were of avian origin and that the isolates were susceptible to antivirals (Kandun et al., 2006). By 2009 researchers had documented 139 incidences of H5N1 infections in Indonesia: 113 were sporadic cases and 26 represented infection clusters. Risk factors for cluster outbreaks were direct exposure to avian viruses. Siblings to index cases were five times more likely to become secondary cases than non‐sibling contacts pointing to a possible genetic contribution to susceptibility for H5N1 infection (Aditama et al., 2011).

### Turkey

In the wake of an epizootic in poultry, a cluster of eight school‐aged paediatric cases of PCR‐confirmed H5N1 infections were observed in Eastern Turkey. All patients were siblings or neighbours and had exposure to chickens which shared living space with them during the cold winter season. All children developed fever and all but one had pneumonia; half of them died. During that time period, four additional isolated H5N1 cases were detected in Turkey. No infections were detected in contacts of cases. Poultry sales in open market were prohibited and 2 million chicken were culled and subsequently, no further cases were observed in Turkey (Oner et al., 2006).

### Antivirals

Ten patients with H5N1 infection were described in the winter 2003 from Vietnam. Patients were young (median age 14 years), coming from eight different places in Vietnam. All but two had direct contact with poultry (holding, killing or defeathering of birds). Seven patients showed diarrhoea in addition to respiratory symptoms. Mortality was extremely high (80%). Two possible secondary cases were reported (Tran et al., 2004). In Vietnam, eight H5N1 patients were treated with the antiviral oseltamivir upon hospitalization for pneumonia. Four patients who survived showed no virus detection after treatment, while in four patients who died, the antiviral did not eliminate the virus. The H5N1 virus from two lethal cases showed the H274Y substitution in the neuraminidase known to confer resistance to this antiviral (de Jong et al., 2005).

### Detailed epidemiology

In Thailand a surveillance program identified 12 H5N1 infections in 2003 associated with a 67% mortality; all occurred in villages that experienced abnormal chicken deaths, nine cases had dying chicken in their backyard (Chotpitayasunondh et al., 2005). Person‐to‐person transmission was documented from one young female who died with H5N1 infection. Her mother had arrived from Bangkok to provide close personal care for her daughter in the hospital. The mother had no contact with poultry. One week later, she developed symptoms and died from H5N1‐confirmed pneumonia a week later. Sequencing revealed an avian‐type H5N1 viral genome displaying an HA gene with avian receptor binding specificity (Ungchusak et al., 2005). A similar case of human transmission of H5N1 infection was reported in China. A father contracted the infection when caring for his adult son who died from H5N1 pneumonia. The father had no exposure to poultry, but close unprotected contact with his hospitalized son. The father developed symptoms, tested positive for H5N1 but survived after receiving oseltamivir. Both cases were infected with an identical avian H5N1 virus showing a polybasic HA cleavage site. Ninety close contacts of the two cases were followed. Two developed mild symptoms but were negative for H5N1 by RT‐PCR. None developed antibodies to H5N1 (Wang et al., 2008). Two children died in Cambodia from H5N1 infection. A seroepidemiological survey was conducted among 670 inhabitants of the two villages where the index cases lived. This region had also experienced a recent H5N1 epizootic in poultry. All tested individuals had close contact with poultry but only 1% of the subjects showed neutralizing antibodies to H5N1. Seropositive subjects were predominantly young and male, but only one subject reported a febrile episode. A parallel case–control analysis revealed bathing in village ponds as a risk factor for H5N1 infections (Vong et al., 2009). In Southeast Asia, H5N1 is endemic in poultry.

### Molecular analysis

An H5N1 isolate from a Vietnamese boy who died in 2014 from an AIV infection was investigated in more detail. The HA was crystallized and its 3‐D structure was analysed. The overall fold resembled more the H1 HA from the pandemic influenza strain of 1918 than that of avian H5 HA yet the receptor binding domain (RBD) resembled closely that of avian H5 HA. Avian H5 HA is constantly evolving and had split into two clades differing by 13 mutations mostly around the RBD. The H5 HA from the Vietnamese boy belonged to the clade 1 Indochina lineage. The receptor binding specificity of this human H5 HA was determined on a glycan microarray and revealed an avian‐typical α‐2‐3 sialyl glycan binding specificity. Binding to a single α‐2‐6‐sialyl‐glycan type was observed that was only expressed in human milk, raising doubt about its relevance as viral receptor. Mutational analysis showed that a shift from the avian to the human receptor specificity identified in the H1 and H3 frameworks of AIVs did not cause an equivalent shift in specificity in the H5 framework with respect to human cell binding (Stevens et al., 2006). The human H5N1 isolates could not be transmitted via aerosol or respiratory droplets between ferrets reflecting the limited human‐to‐human transmission noted by epidemiologists. Virologists investigated to what extent H5N1 isolates modified by site‐directed mutagenesis and submitted to serial passage in ferrets could acquire airborne transmission. Four amino acid substitutions in the RBD of the HA and one in the PB2 protein were necessary for airborne transmission in ferrets (Herfst et al., 2012). For another human H5N1 isolate four HA mutations (N158D/N224K/Q226L/T318I) achieved virus transmission in ferrets. Based on these data, these researchers concluded that only three nucleotide changes separated H5N1 viruses to acquire airborne transmission in ferrets which might, however, not necessary translate into airborne transmission in humans (Imai et al., 2012). Despite using sophisticated mathematical models, researchers stated that estimates of the probability of evolving these mutations cannot be accurately calculated due to persisting knowledge gaps (Russell et al., 2012).

### Replacement by H5N6

Since 2013, the H5N1 group was replaced by H5N6 viruses in birds from Southern China and indeed, in 2014, a first non‐fatal case of a hospitalized H5N6 infection was reported in a 58‐year‐old Chinese who had regularly handled live poultry at a market (Yang et al., 2015). Up to now, 77 cases of H5N6 infections have been reported; most were from China and occurred in 2021 and 2022; 32 infections were fatal (Zhang et al., 2022).

### Tracing HPAI in time and space

An international consortium of virologists reconstructed the path of HPAI H5 infections using epidemiological, geographical and genomic approaches (Xie et al., 2023). Over the last two decades, the HPAI H5 gene has split into 10 phylogenetic clades with clade 2 being the most prominent. In addition, HPAI H5 is associated with different NA subtypes (N2, N6, N8) and acquired new combinations of internal genes. This is in contrast to mammalian influenza viruses which typically maintain a limited number of stable genome constellations. H5 HPAI first established sustained transmission in domestic poultry followed by four important HPAI H5 epizootics in wild birds between 2005 and the present. Until 2017, the H5 epizootics originated in Asia, while in 2020, the H5N8 epizootic emerged in domesticated Egyptian poultry, which spread through the Black Sea route to Eastern Europe. Since mid‐2020 H5N8 was replaced by H5N1 which had acquired in addition to N1 five internal genes from LPAI circulating in European wild birds. H5N1 split into two lineages spreading to North America and Africa respectively. H5N1 has now reached the remote islands of Galapagos and threatens endemic bird species. Also, sea lions and fur seals have been hit hard in South America (Stokstad, 2023). In contrast to earlier HPAI H5 clades which primarily circulated regionally in domesticated birds, the new H5 clade (2.3.4.4b) showed longer persistence in wild than domesticated birds. While the earlier spread of HPAI was dominated by domestic poultry networks and associated human trade activity, the diffusion velocity of the later HPAI was greater in wild birds than in domesticated poultry. The observed changes, namely the shift from HPAI emergence from Asia to Africa and the shift from poultry to wild bird as major driving factor of epizootics, has important consequences. Culling and vaccination, the major control measure to end epizootics in poultry, is not practical in wild birds. Flyways of migratory birds became more important than trade routes for HPAI spread and might be affected by climate change (Cohen, 2023a). Therefore, the control of HPAI transmission across the wild bird‐domestic poultry interface by vaccination (Cohen, 2023b) will become more important to contain HPAI epizootics and potential viral spill‐over into the human population.

## AIV VIRUS ECOLOGY

### Diversity

Influenza A viruses have been isolated from many mammalian species (humans, pigs, horses, mink, felids, marine mammals, bats) and from more than 100 wild bird species belonging to 26 families. Aquatic birds comprising *Anseriformes* (particularly ducks, geese and swans) and *Charadriiformes* (particularly gulls, terns and waders) represent the natural reservoir for influenza A viruses (Olsen et al., 2006). Avian influenza A viruses display HA subtypes H1 to H16 and NA subtypes N1 to N8 and represent thus most of the known HA and NA subtypes relevant in human and veterinary medicine. In wild birds, AIV preferentially infects the intestinal tract which leads to the excretion of large amounts of AIV with the faeces contaminating lake waters where infectious virus persists for days to months depending on ambient temperature conditions. All HA and NA subtypes (exception: H13 to H16) circulate in viruses from ducks of the Northern hemisphere. Ducks show a cyclic pattern of appearance for the different AIV subtypes. Co‐infections with two AIVs as well as cross‐species infection between bird species sharing the same habitat occur naturally.

### Epizootiology

AIV prevalence of up to 60% has been observed in duck populations at their northern breeding grounds but decrease to 2% in the wintering grounds. In wild birds mild respiratory, enteric, or reproductive diseases caused by LPAI viruses are common. As far back as in the 1880s an aggressive disease called fowl plague was described first in Italy affecting mainly domesticated poultry such as chickens, turkeys and geese. Fowl plague cases were until 1959 exclusively caused by H7N7 and H7N1 HPAI viruses. In 1959, a HPAI of the H5N1 type was detected in Scotland from where it spread through Europe, Asia and Africa and reached also the Americas. The HPAI phenotype—so far only associated with H7 and H5 subtypes—is determined by in vivo tests if causing lethal infections in chickens on intravenous inoculation. The HPAI trait correlates genetically with a poly‐basic cleavage site in the HA protein (Lee et al., 2021). Ecological traits were carefully documented for 100,000 LPAI virus samples obtained from wild birds in various parts of the world as well as from 300,000 HPAI virus samples collected on a yearly basis over several decades (Munster et al., 2009). Large surveys showed a dominant role of dabbling ducks (i.e. ducks feeding on vegetable matter on water surfaces) for AIV dissemination. This role is partly explained by their large population size (10 million in Europe alone, half of them mallards) and partly by the fact that a third of these ducks are yearly rejuvenated with young, immunologically naïve birds allowing the circulation of many AIV subtypes. Waders breeding in the artic zones are ecologically important due to their long migration routes spreading AIV over large geographical distances. AIV from gulls do not easily infect ducks under experimental conditions allowing in nature genetic differentiation of AIV by sympatric speciation (i.e. in close geographical proximity). However, also allopatric separation (i.e. by geographical barrier) contributed to genetic differentiation into Eurasian and American AIV lineages. LPAI viruses are generally considered to be non‐pathogenic upon natural infection of wild birds.

### Dispersal by migration

A European study with dabbling ducks investigated whether AIV infection had an effect on their physiology and behaviour. LPAI‐virus‐infected ducks showed a marginally smaller body weight of 20 grams, but infection had no effect on the speed or distance travelled during migration. LPAI infection was associated with 3 days of virus excretion (Latorre‐Margalef et al., 2009). Satellite telemetry of animals fitted with GPS‐collars showed that also H5N2‐infected ducks survived and migrated at least 700 km suggesting potential large‐range dispersal of HPAI viruses by infected wild migratory ducks.

Due to long migration routes of some AIV‐carrying birds (particularly ducks and geese), AIV is widely spread. There are different flyways: along the Pacific coast, the central Mississippi and the Atlantic coast linking North and South America in the New World. In the Old World flyways across Europe, across Central Asia or intercontinental flyways linking Asia with Africa or an eastern route linking Siberia with Australia have been documented. Based on a phylogenetic tree analysis of the viral matrix gene, an American and a Eurasian branch of AIV can be distinguished. The HPAI H5N1 together with the Eurasian swine and Eurasian gull influenza A viruses are distant relatives of the Eurasian avian influenza branch while human influenza A viruses and American swine and American gull influenza A viruses form another group of distantly related influenza A viruses (Olsen et al., 2006).

### Economic impact

In 1997, a HPAI infection with H5N1 was reported on chicken farms and on live bird markets of Hong Kong associated with the first cases of fatal infections of humans with AIV (de Jong et al., 1997). In 2002 H5N1 was detected in waterfowl in parks of Hong Kong and since 2004 H5N1 devastated the poultry industry in large parts of Southeast Asia. Wild bird death from HPAI H5N1 was also observed, first in geese where it killed 10% of *Anser indicus* and subsequently in swans, gulls and herons. In 2006 multiple introductions of distinct H5N1 strains were reported in Nigeria (Ducatez et al., 2006). Migration of wild birds and transport of poultry by trade were considered as likely sources for the H5N1 geographical spread (Normile, 2006).

The economic costs of veterinary H5N1 infections are great: since 2003 it has killed or led to the culling of more than 400 million domestic poultry, causing an estimated $20 billion in financial losses. In 2011, the United Nations Food and Agriculture Organization (FAO) warned of ‘a major resurgence of the H5N1 HPAI with unpredictable risks to human health’. WHO, however, found that H5N1 does not pose an increased risk to public health and that the observed genetic changes are part of the natural evolution of this virus (Normile, 2011).

### Unlinked epidemics

In Bangladesh, human and avian influenza virus epidemiology was investigated between 2010 and 2019 in parallel (Berry et al., 2022). Human influenza virus infection showed marked seasonality with annual peaks in June/July (monsoon season) (in contrast to the marked winter influenza peaks in temperate zones). From 60,000 human respiratory samples, 14% were positive for influenza virus, with 62% representing influenza A and 38% influenza B virus. The dominant influenza A virus was the pandemic A(H1N1)pdm09 strain followed by A(H3) strains. Epidemic waves started in Dhaka metropolitan area and spread radially across the country. Half of 4400 live poultry market samples were positive for AIV predominantly representing H5 AIV followed by AIV H9 subtypes. AIV was present around the year. Reported poultry outbreaks showed seasonality but it was out of phase with human influenza virus epidemics. In Bangladesh, the major human and poultry infection waves are temporally and with respect to HA subtypes not connected.

## CONTAINMENT

### Hygiene measures in markets

A number of interventions reduced the detection rate of AIV in live poultry markets from Asia. The presence of LPAI H9N2 virus was investigated between 1999 and 2011 in live poultry markets from Hong Kong run under different hygiene conditions. Introducing a single rest day per month for cleaning had only a minor effect on the H9N2 detection rate. Excluding the sale of minor poultry such as quail had a greater impact than increasing rest days to two per month. However, the greatest effect (84% reduction of H9N2 detection) was achieved by a ban to keep unsold poultry overnight (Leung et al., 2012). Live poultry markets in Indonesia were investigated between 2009 and 2014: 37% of the samples tested positive for the HPAI virus of H5 subtype. On sight slaughtering increased the risk for a positive sample by fivefold. Including samples from ducks also increased the H5 positivity rate by 5‐fold (Henning et al., 2019). Another study from Indonesia showed that daily waste removal and clear zoning of market activities reduced significantly the rate of the H5N1 detection rate. The presence of ducks on the market was again a clear risk factor (Indriani et al., 2010).

### Markets as infection source

Genome sequencing studies linked H7N9 human infections with live market viral transmission as shown by a case of a Chinese woman working as a butcher on a live poultry market. Epidemiological investigation revealed in her a nearly sequence‐identical virus with that of a poultry‐cage specimen from a neighbouring stall of her working place (Bao et al., 2013). Four patients infected with H7N9 at Zhejiang/China had all contact with poultry 3 to 8 days before disease onset. The viral isolate from one patient was sequenced and turned out to be closely similar to that from an epidemiologically linked chicken at the live market (Chen et al., 2013). Another Chinese study reported on a cluster of H7N9 human cases that were epidemiologically linked to live poultry markets where H7N9 virus was detected. After closure of the markets, no new cases were identified in that region (Han et al., 2013).

### Public health control measures

A number of intervention measures have been introduced outside of live markets to limit the spread of AIV, particularly HPAI viruses, in poultry and thereby to reduce the risk of zoonotic infections in humans. A scientific evaluation of the efficacy of the individual measures is difficult since generally multiple interventions were applied in parallel, frequently without controls. For example, when H5N1 virus was detected in retail markets in Hong Kong in 2002, 22 local farms were identified to be infected. Control was achieved through a combination of quarantine, biosecurity measures, and culling of animals on infected and contact farms resulting in the destruction of about 1 million animals. Finally, animals were vaccinated with a killed H5 AIV vaccine on farms where the infection persisted (Sims et al., 2003). Under these conditions, it is difficult to evaluate the impact of individual interventions (Sims, 2013). The classical approach to H5N1 control is early detection and stamping out of the infection by mass destruction of animals from infected farms. Mass culling worked well in Canada, The Netherlands and Mexico, but H5N1 HPAI virus persisted in Egypt, Indonesia, China, Vietnam and Bangladesh. Resistance from farmers and from people with backyard poultry rearing receiving no financial compensation for culled animals might explain the compromised efficacy of culling in those countries. In areas with low and scattered poultry populations culling might be an ‘overkill’ since HPAI virus infections in those areas might be self‐limiting. Another approach is vaccination. Conducting mass vaccination campaigns is expensive and logistically demanding. The rates of poultry population turnover is high, complicating the achievement of herd immunity levels that can interrupt virus transmission. Further problems are the timing of vaccination (interference with maternal antibodies in young birds), numbers of doses (one dose is insufficient in ducks) and the quality of the vaccine in connection with antigenic match, antigen content, adjuvant use and cold storage problems. Biosecurity measures are difficult to implement in countries where poultry are not reared on large farms but in backyards, in buildings and rooftops, as free‐grazing ducks or scavenging chickens in villages.

### Resistant poultry

Since sterile immunity cannot easily be achieved with AIV vaccination in birds, researchers have explored alternative ways to decrease transmission of AIV in poultry. They constructed transgenic chickens that expressed an RNA hairpin molecule containing a mutated complementary RNA (cRNA) binding site capable of inhibiting influenza viral polymerase activity by a decoy mechanism. Although transgenic chickens were not protected against infection with AIV, they were unable to transmit the virus even to fully susceptible, non‐transgenic control chickens (Lyall et al., 2011). The goal of this research was a proof‐of‐concept demonstration. Introduction of such resistant animals into chicken breeds by large animal providers might theoretically be technical feasible in industrialized countries (but might meet strong opposition against GMO animals in the human food chain) but such a GMO approach will not reach backyard chicken rearing in developing countries where contact between chicken and humans is the closest (Enserink, 2011). Recently, AIV‐resistant chicken were also created by genome editing of host proteins interacting with the AIV polymerase (Idoko‐Akoh et al., 2023) (see below Host factors cooperating with viral polymerase: ANP32).

## MOLECULAR TRAITS OF THE SPECIES BARRIER: TARGETS FOR A SEQUENCE‐BASED EARLY WARNING SYSTEM?

### Risk models versus sequence analysis

Early warning systems for animal virus cross‐infections are a crucial part of pandemic preparedness. As influenza viruses have repetitively caused pandemics in the human population, they are likely or at least possible candidates for the next pandemic—hence, the importance of risk models for assessing the likelihood of different animal influenza viruses as causative agents for such a pandemic. However, the authors of both the CDC IRAT and WHO TIPRA risk models insist that prediction of the virus causing the next influenza pandemic is currently impossible. In fact, the high scores given to avian H7N9 and H5N1 influenza A viruses by both models, which are also the focus of the present review, are influenced by prior outbreaks with these virus types in the human population. These models therefore represent to a certain degree more a retrodiction of what has already happened in the past than predictions of what might occur in the future. In addition, IRAT and TIPRA depend on many medical, veterinary, virological and epidemiological survey data that are laborious, costly and sometimes dangerous to obtain. The question arises whether the now rapidly accumulating sequencing data on viral genomes and metagenomes could be exploited for a purely sequence‐based early warning system for pandemic risk assessment. The large database on influenza virus genomes (GISAID), systematic worldwide virus sampling efforts currently in place and the wealth of data on influenza virus host interaction and past pandemics make sequence‐based early warning worth exploring.

Early warning by sequence analysis for avian influenza viruses could rely on genetic definitions of the viral species barrier between birds and mammals. Basically, the efficacy of such an early warning system hinges on the knowledge about factors that limit a virus in infecting a new host species.

### HA cleavage site

Several steps in the influenza replication cycle meet barriers. Not all barriers determine what species is infected, some determine what organs are infected, sometimes with important consequences for the virulence of the viral infection and therefore also of pandemic impact. An example is the activation of the HA protein from influenza viruses by proteolytic cleavage. The fusion of the viral with the endosomal membrane which is essential for the release of the viral genome into the cytoplasm of the infected cell is mediated by the HA protein. Fusion activity is induced by the proteolytic cleavage of the HA0 precursor protein into the subunits HA1 and HA2 which exposes the fusion peptide. The difference between a mono‐basic and multi‐basic HA cleavage site (CS) determines the virulence of AIV in poultry which, as mentioned earlier, differentiates LPAI from HPAI viruses. The difference is explained by the substrate specificity of host proteases. Mono‐basic CS are cleaved by trypsin‐like proteases expressed by respiratory and intestinal cells in poultry. Multi‐basic CS (i.e. containing more than one basic aa residue at the cleavage site) in contrast can be proteolytically processed by the ubiquitously expressed furin‐like proteases allowing the infection of endothelial vascular cells by AIV and thus systemic viral spread in poultry and severe veterinary infections. Paradoxically, ducks do not suffer severe infections with HPAI from poultry which was explained by high expression of the natural NRP1 ligand Sma3a on endothelial cells which blocks binding of HPAI virus to the NRP1 cell receptor and thereby prevents viral uptake in endothelia of ducks. Most human influenza viruses possess a mono‐basic CS and for them type‐II transmembrane serine protease 2 (TMPRSS2) has been identified as a major activating protease on human airway cells (Heindl & Böttcher‐Friebertshäuser, 2023). While a multi‐basic HA cleavage site is a warning sign, introduction of a multi‐basic CS into low pathogenic H5N1 or into human H3N2 virus in isolation did not create highly pathogenic virus variants. This observation underlines that a multi‐basic CS is a necessary, but not sufficient basis for high pathogenicity (Neumann et al., 2021).

### Viral receptors

Influenza viruses bind to sialic acid‐containing glycans expressed on the cell surface as viral receptors. The human upper respiratory tract and the trachea contain α‐2,6Gal (galactose) sialyloligosaccharides, but not α2,3Gal sialyloligosaccharides on the cell surface. Human influenza viruses therefore bind α‐2,6Gal, but not α2,3Gal sialylglycans. In contrast, AIV preferentially bind α2,3Gal sialyloligosaccharides which are expressed on the surface of respiratory and intestinal epithelia in birds. This difference defines a strong species barrier for cross‐infection of humans by avian influenza viruses. In contrast, pig trachea epithelial cells contain both α2,6Gal sialyl and α2,3Gal sialylglycans and can therefore be infected by both avian and human influenza viruses. This observation explains why pigs are epidemiologically a mixing vessel for the creation of reassortant human‐avian influenza viruses in pigs which are then potentially preadapted for transmission into the human population as seen in the 2009 Swine flu pandemic with the A(H1N1)pdm09 strain. The molecular basis of receptor recognition by HA has been intensively investigated. For avian H2 and H3 HA proteins changes in aa positions 226 and 228 confer Sialo (Sia)α2,6Gal binding specificity to avian viruses. For H1 HA changes at aa positions 190 and 225 allowed AIV binding to both Siaα2,6Gal and Siaα2,3Gal glycans (Neumann et al., 2021). In H5 HA a double mutation at aa positions 226 and 228 also showed substantially reduced affinity to Siaα2,3Gal glycans and increased binding to biantennary N‐linked glycans with α2,6–linked sialic acids. Three mutations in H7 HA at aa positions 186, 193 and 228 changed the receptor specificity from Siaα2,3 to Siaα2,6 glycans (Neumann et al., 2021). The corresponding changes in AIV field isolates could thus serve as a sequence‐based warning signal for cross‐species infection risk. However, the researchers differed in their view with respect to the predicting power of such sequence changes. While in an earlier publication, the authors cautioned that knowledge of genetic changes in circulating viral isolates by themselves cannot be used to predict the impact on receptor specificity (Stevens et al., 2006), scientists in a more recent publication were more optimistic but they claimed that deep sequencing is needed to detect rare variant viruses presenting signatures pointing to host range extensions (Russell et al., 2012).

### RNA polymerase mutations

Species barrier traits for infections with avian and human influenza viruses were also defined for later steps of virus replication. For example, a single gene reassortant virus which derives its PB2 gene from an avian virus and all remaining genes from a human virus showed a host range restriction phenotype. It replicated efficiently in avian cells but failed to replicate on MDCK, a reference mammalian cell line for propagation of human influenza viruses. Upon selection on MDCK cells, four mutants could be recovered that replicated efficiently on the MDCK cell line. All showed a single aa change at position 627 of PB2. At this point in time, all known isolates of avian viruses encoded glutamic acid (E in one letter code) and all human viruses lysine (K) at this position (Subbarao et al., 1993). Ten years later, 19 natural avian virus isolates also displayed a lysine at this position and all belonged to the HPAI group of H5N1 (notably including isolates from a spectacular die‐off of waterfowl at the Chinese Qinghai Lake) and H7N7. This mutation in H5N1 was also linked with high pathogenicity in mice (Taubenberger et al., 2005). Apparently, this mutation permits escape from a species‐specific restriction factor that targets polymerases from avian viruses in mammalian cells. Paradoxically, the swine influenza H1N1 strain from the 2009 pandemic has a glutamic acid at the 627 position of PB2. Researchers subsequently determined that the pandemic H1N1 had second‐site mutations in PB2 that allowed escape from this restriction in mammalian cells and this new aa constellation was also effective when transferred to typical AIV. These second‐site mutations are widely distributed in PB2 from swine influenza viruses. This observation was interpreted as pre‐adaptation to introgression into the human population (Mehle & Doudna, 2009). It is likely that further research into to molecular basis of species barrier mechanisms will identify further critical positions in the viral genome that allow early warning for avian influenza viruses that have acquired mutations facilitating cross‐species infections.

### Host factors cooperating with viral polymerase: ANP32

Host factors limiting the replication of heterologous influenza viruses have also been identified. For example, a screen of chicken genome radiation hybrid cells identified a single chicken gene, ANP32A, that supports AIV polymerase activity when expressed in human cells. ANP32A is a nuclear protein that is implicated in transcriptional regulation of the host. As a co‐opted function, it helps to increase levels of viral genomic RNA (vRNA) and complementary antigenomic RNA (cRNA) synthesis in an in vitro polymerase assay (Long et al., 2016). In nature, AIV has co‐opted avian ANP32A as a host factor for its replication and cannot use the shorter mammalian ANP32A homologue. Expression of the chicken ANP32A in human cells allowed replication of AIV. Replication of the viral genome requires the formation of a replicative platform consisting of two heterotrimeric polymerase molecules (comprised of PB1, PB2 and PA proteins) bridged by ANP32A. Chicken ANP32A variants containing either the N129I or D130N amino acid substitutions failed to complement AIV polymerase function in human cells (Long et al., 2019). Using CRISPR/Cas9 genome editing (GE), chicken were created that carried mutant ANP32A with these mutations. GE chicken were indeed resistant to low doses of AIV infection and failed to transmit the virus to control chicken. Upon high AIV dose exposure, the GE chicken showed a low level of breakthrough infections displaying PA‐E349K, PA‐T639I or PB2‐M631L mutations. Two of these mutations have also been identified in AIV infecting humans (Hatcher et al., 2017) suggesting that they are human‐adaptive mutations. The escape AIV mutants had co‐opted alternative ANP32 protein family members, ANP32B and ANP32E. Additional genome editing for removal of ANP32B and ANP32E eliminated all viral growth in chicken cells but such extensive genome editing is likely to adversely affect the health of the chicken (Idoko‐Akoh et al., 2023).

### Escape from innate immunity: MxA

Viruses do not only depend on host gene functions to achieve their replication, viruses must also escape innate immunity if they want to be efficiently transmitted in the human population. A German‐Chinese consortium of researchers argued that there must be susceptibility genes for cross‐infection with AIV based on the observation that many subjects are exposed to AIV but only few get infected. To substantiate their hypothesis they conducted a case–control study with 220 H7N9‐infected patients and 120 epidemiologically linked healthy poultry workers. Whole genome sequencing demonstrated that carriers of rare single nucleotide variants (SNV) in the gene *MX1* were 6‐fold more likely to be H7N9 AIV infected. *MX1* encodes the myxovirus resistance protein A (MxA). MxA inhibits AIV polymerase activity. Twelve of the 15 SNV of MX1 caused a loss of this antiviral activity. MxA has a negative‐dominant effect by forming nonfunctional MxA hetero‐oligomers. Apparently, severe disease can be caused by inborn errors of immunity in the host. The researchers predicted that zoonotic AIVs have to acquire adaptive mutations, predominantly in the viral nucleoprotein (NP), that allow some degree of MxA escape (Chen et al., 2021).

### Escape from innate immunity: BTN3A3

Type I interferon (INF) response is one major element of the host antiviral innate immune mechanisms. Researchers expressed more than 800 IFN‐stimulated genes and screened them for antiviral activity against a panel of influenza viruses. BTN3A3 (butyrophilin subfamily 3 member A3) inhibited the replication of the avian (Mallard) influenza virus by 100,000‐fold, but not that of human influenza viruses. Knockdown of BTN3A3 by small‐interfering RNA restored the replication of AIV. The inhibitory BTN3A3 activity is specifically directed against AIV (no other human respiratory viruses were inhibited), evolved in primates and BTN3A3 is constitutively expressed in human airways. It acts at the early stages of the virus replication cycle by inhibiting AIV RNA replication. The researchers then studied reassortant Mallard AIV and associated gene segment 5 encoding the viral nucleoprotein (NP) with sensitivity towards BTN3A3 both in cell culture and in mice. Avian and human NPs differ at 7 aa positions, five positions were previously also associated with avian‐to‐human transmission events. Replacement of the avian‐typical by a human‐typical aa at the position 313 (single F313Y mutation) allowed the Mallard virus to evade BTN3A3 inhibition. Interestingly, avian H7 and H9 serotypes that succeeded to infect humans also evaded BTN3A3 restriction. They maintained the avian‐typical aa at position 313, but mutated at position 52, that is adjacent to residue 313 in the 3‐D NP structure. NP position 52, but not 313 was also implicated in evasion from MX1. Overall, 77% of the 1700 AIV isolated from humans showed the BTN3A3‐resistant genotype. Over the last 50 years, different serotypes of AIV in bird populations showed transient peaks of the BTN3A3‐resistant genotype that temporally correlated with the timing of the spillover events into the human population. In contrast, very few of these AIV showed the ‘mammalian’‐adapted PB2 at aa position 627.

## MINK FARMS AND LIVE ANIMAL MARKETS AS CRITICAL CONTROL POINTS

Research has provided an increasing list of viral point mutations that define viral virulence and transmission potential genotypically. The speed and low cost of viral genome sequencing speak in favour of exploiting this knowledge for influenza pandemic preparedness efforts. The importance of such efforts is underlined by a recent outbreak of HPAI H5N1 virus on a large mink farm in Galicia/Spain in October 2022 (Agüero et al., 2023). Epidemiological analysis suggested that mink‐to‐mink transmission had occurred on the farm and that H5N1 virus from seabirds of the Galician coast was the most likely infection source. Genomic analysis revealed a mink viral strain closely related to an H5N1 gull isolate from France isolated in 2022. From this gull viral strain, the mink viral strain differed by only 9 aa changes including one at position 271 of PB2 (T271A). This mutation was previously shown to enhance the viral growth in mammalian cells and to increase the viral titre in the lungs of mice (Bussey et al., 2010). This mutation was also detected in the avian‐derived PB2 of the 2009 pandemic H1N1 influenza virus. The back‐mutation from alanine (A) to threonine (T) at position 271 of PB2 also abolished the virus' respiratory droplet transmission in guinea pigs (Zhang et al., 2012). Minks are reared on big farms for fur production. They can be infected with both avian and human influenza viruses and can thus—similar to pigs—serve as mixing vessels for the generation of pre‐adapted influenza viruses from which pandemic strains could spill over into the human population. Viral genome sequencing of influenza outbreaks on affected mink farms will thus be important to assess to what extent genomic traits for mammalian transmission were acquired. Likewise, influenza viral sequencing in poultry from live markets and in wild birds suffering increased death rates will be essential to assess whether pre‐adapting mutations for mammalian transmission were observed.

No worker on the Galician mink farm got infected. This might be due to the fact that after the first identification of SARS‐CoV‐2 infection in mink farms in the Netherlands (Lu et al., 2021), the use of a face mask was made compulsory for all farm workers on mink farms in Spain. Therefore in addition to diagnostic sequencing, hygiene barriers should be introduced on mink farms including measures preventing the access of wild birds to the mink cages. Live animal markets make also logical targets for viral sequencing efforts as part of an early warning system to alert to influenza viruses pre‐adapted for crossing into and for spreading in the human population.

## AUTHOR CONTRIBUTIONS

**Harald Brüssow:** Conceptualization (equal); writing – original draft (equal); writing – review and editing (equal).

## CONFLICT OF INTEREST STATEMENT

The author declares no conflict of interest.
